# Effects of rainfall amount and frequencies on soil net nitrogen mineralization in Gahai wet meadow in the Qinghai-Tibetan Plateau

**DOI:** 10.1038/s41598-023-39267-3

**Published:** 2023-09-08

**Authors:** Guorong Xu, Guang Li, Jiangqi Wu, Weiwei Ma, Haiyan Wang, Jianyu Yuan, Xiaodan Li

**Affiliations:** 1 College of Forestry, Gansu Agricultural University, Lanzhou, 730070 China; 2 State Key Laboratory of Aridland Crop Science, Gansu Agricultural University, Lanzhou, 730070 China; 3 College of Resources and Environmental Sciences, Gansu Agricultural University, Lanzhou, 730070 China

**Keywords:** Climate sciences, Ecology

## Abstract

Global climate change has led to a significant increase in the frequency of extreme rainfall events in the Qinghai-Tibetan Plateau (QTP), thus potentially increasing the annual rainfall amounts and, consequently, affecting the net soil nitrogen (N) mineralization process. However, few studies on the responses of the soil net N mineralization rates to the increases in rainfall amounts and frequencies in alpine wet meadows have been carried out. Therefore, the present study aims to assess the effects of rainfall frequency and amount changes on the N fixation capacity of wet meadow soils by varying the rainfall frequency and amount in the Gahai wet meadow in the northeastern margin of the QTP during the plant-growing season in 2019. The treatment scenarios consisted of ambient rain (CK) and supplementary irrigation at a rate of 25 mm, with different irrigation frequencies, namely weekly (DF1), biweekly (DF2), every three weeks (DF3), and every four weeks (DF4). According to the obtained results, the increased rainfall frequency and amount decreased the soil mineral N stock and increased the aboveground vegetation biomass (AB) amounts and soil water contents in the wet meadows of the QTP. Ammonium (NH_4_^+^-N) and nitrate N (NO_3_^–^-N) contributed similarly to the mineral N contents. However, the ammonification process played a major role in the soil mineralization process. The effects of increasing rainfall amount and frequency on N mineralization showed seasonal variations. The N mineralization rate showed a single-peaked curve with increasing soil temperature during the rapid vegetation growth phase, reaching the highest value in August. In addition, the N mineralization rates showed significant positive correlations with soil temperatures and NH_4_^+^-N contents and a significant negative correlation with AB (*P* < 0.05). The results of this study demonstrated the key role of low extreme rainfall event frequencies in increasing the net soil N mineralization rates in the vegetation growing season, which is detrimental to soil N accumulation, thereby affecting the effectiveness of soil N contents.

## Introduction

Nitrogen (N) is one of the main ubiquitous nutrient elements in ecosystems, playing a limiting role in plant growth^1,2^. In terrestrial ecosystems, soil N availability to plants is highly dependent on soil N mineralization, which is the key process controlling the global N cycle^3^. Indeed, soil microorganisms can transform insoluble organic N into soluble organic N through the action of extracellular enzymes, then into inorganic N forms that plants can absorb and utilize^4,5^. Soil N mineralization is the key process transforming organic N into inorganic N, namely ammonium N (NH_4_^+^-N) and nitrate N (NO_3_^–^-N), through the ammonification and nitrification processes, which are the major internal ecological processes influencing the availability of soil inorganic N to plants and microorganisms^6,7^. Net N mineralization refers to the net accumulation of inorganic N in the soil solution over a specific period^8^. Absorption and chemical fixation are the main processes by which microorganisms fix inorganic N from soils^9^. Soil N availability is limited by inorganic N supply rates and controlled by N mineralization and fixation, making the net N mineralization rate an important indicator for evaluating N effectiveness^10^.

Wetland ecosystems are interstitial zones between the terrestrial and aquatic ecosystems, characterized by high species diversity and productivity. These ecosystems play, in fact, an important role in the global N cycle. The distinct soil and water interface and high biological productivity are conducive to rapid N cycle transformations in wetland ecosystems^11^. Indeed, several researchers have devoted considerable attention to N storage and transformation in wetland ecosystems. Previous studies have shown that extreme weather events are susceptible to be more frequent in the future due to global climate change, thereby strongly affecting the global water cycle^12–15^. Changes in the global water cycle can affect short-term soil N mineralization and, consequently, affect plant nutrient uptakes^16,17^. Soil water is one of the main factors affecting net soil N mineralization. In fact, precipitation changes can directly affect soil water contents, thus affecting soil N effectiveness and N mineralization rates^18^. Previous studies have highlighted positive correlations between soil water contents and soil N mineralization rates within a certain range^19^. Indeed, soil net N mineralization and nitrification rates may be enhanced with increasing soil water contents, thereby increasing soil inorganic N contents and enhancing the effectiveness of soil N^20,21^. However, some studies have pointed out decreases in soil N mineralization and nitrification rates at high soil water contents, thus enhancing N loss in the form of gaseous N through the denitrification pathway^22,23^. In addition, it was demonstrated that frequent wet-dry alternations promote the decomposition of soil organic matter, thus significantly increasing soil mineralization rates^24^. To date, most related studies have assessed the effects of soil water contents on N mineralization using indoor incubation experiments by simply regulating soil water contents. However, few studies have investigated soil N mineralization through in-situ incubation experiments under natural rainfall changes. Indeed, it is necessary to assess the response mechanisms of soil inorganic N contents and N mineralization rates under different rainfall frequencies to better understand the soil N transformation processes in wet meadows.

As the highest and largest plateau in the world, the QTP is an ecologically fragile area sensitive to global climate change^25^. Indeed, several studies have highlighted increasing trends in air temperatures and precipitation amounts in the QTP over the past decade due to climate change^26–28^. In addition, actual global climate change models predicted continuous increases in the frequency and magnitude of extreme rainfall events in the QTP^29^. Precipitation and frequency changes in highland ecosystems can directly lead to changes in soil moisture contents, resulting in potential frequent soil wet-dry alternations and, consequently, affecting soil N mineralization rates and limiting alpine wet meadow production^11^. Numerous studies have investigated the effects of warming and N additions on soil N mineralization rates in alpine wet meadows, while only a few studies have assessed the response of soil N mineralization rates to extreme rainfall frequencies in alpine wet meadows. Previous related studies have shown that the soil N mineralization rates in the wet meadows of the QTP are closely related to soil temperatures and soil moisture contents. In fact, temperature is the main driving factor, while soil moisture content is an important determinant of N mineralization rates in alpine wet meadows^12^. Although alpine wet meadow ecosystems are characterized by high soil water contents, the role of soil water contents in the N cycle is often overlooked or considered less important in the context of changing rainfall amounts and frequency. Moreover, the mechanisms involved in the effects of soil water content changes on N transformation processes in wet meadow ecosystems under climate change remain unclear. Therefore, further studies on the responses of soil N mineralization rates in alpine wet meadows to the changes in rainfall amounts and frequency are required.

In this context, we conducted a field experiment to assess the effects of rainfall frequency changes on soil N mineralization rates in the Gahai wet meadow of the QTP. Our specific research objectives were to: (1) further discuss the response mechanisms of N mineralization rates to the increases in the rainfall frequency and amount; (2) investigate the effects of the increase in the rainfall frequency on the N fixation capacity of the wet meadow soil. The following hypothesize were formulated in this study: (a) The increase in the rainfall frequency increases the soil water contents and decreases the soil air contents in the study area, thereby reducing the N mineralization rates; (b) The increase in the rainfall frequency promotes soil N leaching and, consequently, decreases soil inorganic N contents and N fixation capacity. To test these hypotheses, five rainfall frequencies were considered in this study based on rainfall amount and frequency monitoring data in the Gahai wetlands. The net N mineralization rates, inorganic N contents, water contents, and temperature of the wet meadow field soil were determined under different rainfall frequencies.

## Materials and methods

### Study area

The present study was conducted in the Gahai Zecha National Nature Reserve in the eastern part of the QTP (33°58′12″–34°32′16″N, 102°05′00″–102°47′39″E), which is the intersection of the Yellow and Yangtze rivers. The QTP is a water conservation and important transit area, as well as a habitat and breeding place for North–South migratory birds. Indeed, the study area has been listed among internationally important wetlands. The wet meadow of the study area covers a total area of 57,846 ha, accounting for 88.4% of the total wetland area. The region is characterized by a cold-temperate continental monsoon climate. The average temperature in the hottest (July) and coldest (January) months are 10.5 and − 8.5 °C, respectively, with an average annual temperature of 2.9 °C. There is no absolute frost-free period in the study area. On the other hand, the average annual rainfall is 785 mm, of which 76% of the total annual rainfall amount occurs mainly in the May–September period. In addition, the highest rainfall amounts occur mainly in August. Affected by the climatic conditions of the plateau, mountain types, and the freeze–thaw effect, the soil types in the region consist mainly of meadow, bog, and peat soils. The field plot considered in this study is characterized by meadow soil, with sandy loam and clayey soil textures in the 0–20 and 20–40 cm soil layers, respectively. The main plant species in the study area consist of *Carexmeyeriana*, *Thalictrum aquilegifolium*, *Artemisia subulata*, *Potentilla chinensis*, *Polygonum viviparum L*., and *Potentilla anserina L*^28,30^.

### Experimental design

In this study, a typical wet meadow field was selected in 2019 in the Gahai-Zecha wetland. We established a total of 15 experimental plots, covering a total area of 100 m^2^. The experimental design consisted of five treatments with three replications. Each plot covered 4 m^2^ (2 m × 2 m), with 2 m spacing as a buffer zone. The extreme precipitation event in the Tibetan Plateau was about 25 mm·d^–1^, of which the frequency did not exceed five times per year^31^. However, the frequency of the extreme precipitation event is expected to increase significantly in the future, thus potentially increasing the precipitation amounts^32^. Extreme rainfall events were mainly concentrated during the plant growing season^27,29^. In this study, we used sprinklers to slowly irrigate each plot with the collected rainwater from May to early October 2019, ensuring even infiltration of the rainwater at each plot without generating surface runoff^29^. In total, four different rainfall frequency events were considered in this study to simulate precipitation inputs to the field experiment area based on an irrigation rate of 25 mm, taking into account ambient rainfall as a control (CK)^28,33^. The irrigation frequency treatments with natural rainwater consisted of one watering per week, 2 weeks, 3 weeks, and 4 weeks, corresponding to total irrigation amounts of 475 mm (DF1) (19 times × 25 mm), 225 mm (DF2) (9 times × 25 mm), 150 mm (DF3) (6 times × 25 mm), and 100 mm (DF4) (4 times × 25 mm), respectively. The first irrigation event occurred on May 27, 2019.

### Measurement of soil nitrogen mineralization

In-situ incubations can reflect the actual states of soil net N mineralization, nitrification, and ammonification^34,35^. In this study, we used the in-situ closed-top PVC tube incubation method to ensure the same structure and temperature characteristics of the experimental soil as the external soil^36^. At the beginning of the growing season, two PVC pipes with an inner diameter of 5 cm were placed vertically in each square of the soil at a 20 cm depth. In addition, a soil core sample was collected from the experimental field to determine the initial soil NH_4_^+^-N and NO_3_^–^-N contents, while other soil cores were in-situ incubated after sealing the topsoil with a plastic film to prevent rainwater infiltration and to exclude vegetation roots while allowing gas exchange. The remaining PVC pipes and soil samples were collected after 15 days of in situ incubation. New PVC tubes were placed in each soil square following each growing period to ensure continuous in-situ observations. Soil samples were collected from the 0–10 and 10–20 cm soil layers. Each treatment was repeated 3 times. All soil samples (initial and incubated) were hand sorted and sieved through a 2 mm sieve. The incubated soil samples were processed in the same manner as the initial cores. All initial and incubated soil samples were sent to the laboratory and analyzed within 24 h^35^.

### Aboveground vegetation biomass

The aboveground vegetation biomass (AB) was determined in this study based on the cumulative plant biomass in August 2019, corresponding to the peak of the plant growing season. Three 25 cm × 25 cm squares were first randomly selected from each treatment plot, then plants were harvested from the squares to determine the AB. The collected biomass samples were initially oven-dried at 100 °C for 0.5 h, then over-dried at 60 °C for 72 h until reaching constant weights^35^. The dry weights of the biomass samples were measured using analytical balances.

### Soil water contents and soil temperatures

In this study, soil water contents (SWC) and soil temperatures (ST) at the 0–10 cm and 10–20 cm depths were measured in situ using a soil moisture content analyzer (QS-SFY/RS232), Qiang Sheng Manufacturing Center of Analysis Instruments, Beijing, China) and temperature sensor, respectively, during soil sampling^35^.

### Determination of soil carbon and N forms

#### Determination of soil NH_4_^+^-N and NO_3_^–^-N contents

Approximately 10 g of the collected soil samples were first added to 50 mL of 2 M KCl solutions, then mixed for 1 h using a rotational shaker. Soil NH_4_^+^-N and NO_3_^–^-N were separated by distillation using magnesium oxide (MgO) and Devarda’s alloy (50% Al, 45% Cu, and 5% Zn)^28,30,33^. Indeed, parts of the soil solution samples were steam-distilled with MgO using a steam distillation system to separate soil NH_4_^+^-N, while the remaining parts in the flasks were redistilled after adding Devarda’s alloy and 1 mL sulfamic acid to separate soil NO_3_^–^-N. The released NH_3_ was trapped using a boric acid solution, then converted into (NH_4_)_2_SO_4_ using 0.05 mol·L^–1^ H_2_SO_4_ solution. The NH_4_^+^-N and NO_3_^–^-N contents were determined based on the volume of sulfuric acid added^36–38^.

#### Determination of soil dissolved organic N (DON) contents

The DON contents were determined according to the method described by Willett^39^. Specifically, 10 g fresh soil samples were placed into 150 mL Erlenmeyer flasks, then 50 mL of 0.5 M K_2_SO_4_ solutions was added and shaken at 200 rpm for 1 h. The mixtures were centrifuged at 12,000 rpm for 10 min. 5 mL of the solutions were used to determine the total dissolved N contents using the Semi-Micro Kjeldahl method. The DON content was determined by calculating the difference between the total dissolved N contents and the combined NH_4_^+^-N and NO_3_^–^-N contents^39^.

#### Determination of soil microbial biomass N (MBN)

The MBN contents in the soil samples were determined using the chloroform fumigation extraction method^40^. Two groups of 10 g fresh soil samples were placed in a vacuum desiccator. The first group was fumigated with ethanol-free chloroform for 24 h, while the second group was considered the control group (without fumigation). The fumigated and unfumigated soil samples were mixed with a 0.5 M K_2_SO_4_ solution and shaken at 180 rpm for 30 min using a shaker. 5 mL of the supernatants were extracted and analyzed using the semi-micro Kjeldahl method^41–44^:

$${\text{Cfumigated }} - {\text{ Cnonfumigated}}/0.{38},$$

where Cfumigated and cnonfumigated denote the MBN contents in the fumigated and unfumigated soil samples, respectively.

#### Determination of soil bulk density (BD), soil total nitrogen (TN) contents, and soil total phosphorus (TP) contents

In this study, some undisturbed soil samples were collected using a ring knife, then oven-dried at 105℃ for 24 h to determine the soil BD values^45^. On the other hand, 1 g of the air-dried soil samples were nitrified using concentrated H_2_SO_4_ at 400℃ until achieving a milky white color. The solutions were placed then into 100 mL volumetric flasks, then 5 mL and 10 mL of the solutions were analyzed for the TN and TP contents using the Semi-Micro Kjeldahl and molybdenum colorimetric methods, respectively^46,47^.

#### Determination of soil organic carbon (SOC) contents

The SOC contents in the collected samples were analyzed in this study using the improved Walkley and Black method. Specifically, 0.2 g (accurate to 0.001 g) of the air-dried soil samples were digested at 180 °C for 30 min using 5 ml of 0.8 M 1/6K_2_CrO_7_ and 5 ml of concentrated H_2_SO_4_. Afterward, 2–3 drops of Ortho phenanthroline indicator were added to the solutions, followed by titration with standardized FeSO_4_. The SOC contents were determined based on the consumed K_2_CrO_7_^48^.

### Data calculation

According to the difference in the soil NH_4_^+^-N and NO_3_^–^-N contents before and after culture, the net ammonification, net nitrification, and net N mineralization rates of the soil were calculated in mg·kg^–1^ using the following formulas^49–51^:

$$\Delta {\text{t }} = {\text{ ti}} - {\text{t}}0,$$

$$\Delta {\text{c }}\left( {{\text{NH}}_{4}^{ + } - {\text{N}}} \right) \, = {\text{ c }}\left( {{\text{NH}}_{4}^{ + } - {\text{N}}} \right){\text{ ti}} - {\text{c }}\left( {{\text{NH}}_{4}^{ + } - {\text{N}}} \right){\text{ t0}},$$

$$\Delta {\text{c }}\left( {{\text{NO}}_{3}^{ - } - {\text{N}}} \right) \, = {\text{ c }}\left( {{\text{NO}}_{3}^{ - } - {\text{N}}} \right){\text{ ti}} - {\text{c }}\left( {{\text{NO}}_{3}^{ - } - {\text{N}}} \right){\text{ t}}0,$$

$${\text{Ra }} = \, \Delta {\text{c }}\left( {{\text{NH}}_{4}^{ + } - {\text{N}}} \right)/\Delta {\text{t,}}$$

$${\text{Rn }} = \, \Delta {\text{c }}\left( {{\text{NO}}_{3}^{ - } - {\text{N}}} \right)/\Delta {\text{t,}}$$

$${\text{Rm }} = \, (\Delta {\text{c }}\left( {{\text{NH}}_{4}^{ + } - {\text{N}}} \right) + \Delta {\text{c }}\left( {{\text{NO}}_{3}^{ - } - {\text{N}}} \right))/\Delta {\text{t}},$$

where Δt denotes the time interval before and after culture; Δc (NH_4_^+^-N) denotes the change in the soil NH_4_^+^-N contents before and after culture; Δc (NO_3_^–^-N) denotes the change in the soil NO_3_^–^-N contents before and after culture; Ra, Rn, and Rm denote the net ammonification, net nitrification, and net N mineralization rates, respectively, of the soil.

### Statistical analysis

In this study, the one-way ANOVA analysis was performed to determine whether the soil inorganic N contents, Ra, Rn, and Rm were statistically different between different sampling dates and treatments (at the P < 0.05 level). All the collected data were assessed for variance homogeneity and normal distribution. The effects of soil depths and rainfall frequency, as well as their interactions, on the Rm values, were assessed using the two-way ANOVA analysis. In addition, a regression analysis was performed to analyze the relationship between soil water contents, soil temperatures, and soil net N mineralization rates. All statistical analyses were performed using SPSS 20.0 software (SPSS for Windows, Chicago, USA), taking into account the significance level of P < 0.05. Whereas graphs were generated using Origin 2017.

## Results

### Soil properties and aboveground vegetation biomass

The obtained results showed different effects of rainfall frequencies and amounts on soil nutrients (Table 1). Indeed, the soil BD, TN contents, TP contents, and DON contents decreased significantly with increasing rainfall frequency and amount (P < 0.05). However, significantly lower DON contents were observed in DF1 and DF4 than those in CK (P < 0.05). In contrast, no significant differences in the DON contents were observed between DF2 and DF3 (P > 0.05). The SOC contents in DF2 were significantly higher than those in CK, while the SOC contents in DF3 and DF4 were significantly lower than those in CK (P < 0.05). The soil MBN contents in the DF1 and DF3 treatments were significantly higher than those in CK. In addition, the increase in the rainfall amount and frequency significantly increased the AB amounts in DF1, DF2, and DF3 compared to those in CK (P < 0.05) (Table 1).

**Table 1:** The main physicochemical indicators of the soil under different treatments.

	TP (mg·kg^–1^)	TN(g·kg^–1^)	SOC(g·kg^–1^)	DON (mg·kg^–1^)	MBN (abs·g^–1^)	AB(g·m^–2^)	BD(g·cm^–3^)
CK	60.25 ± 0.85A	2.99 ± 0.09A	29. 83 ± 0.20B	48.24 ± 2.77A	0.08 ± 0.00B	85.82 ± 2.69D	1.41 ± 0.00A
DF1	47.88 ± 0.58C	2.48 ± 0.02B	29.96 ± 0.31B	38.47 ± 1.67B	0.10 ± 0.00A	245.79 ± 12.92A	1.02 ± 0.02D
DF2	55.30 ± 3.87AB	2.38 ± 0.03B	32.37 ± 0.51A	48.00 ± 2.00A	0.07 ± 0.00C	173.36 ± 5.94B	1.19 ± 0.01C
DF3	53.31 ± 0.22BC	2.16 ± 0.02C	28.80 ± 0.23C	46.01 ± 2.23A	0.10 ± 0.00A	139.26 ± 8.81C	1.32 ± 0.02B
DF4	52.63 ± 0.33BC	2.31 ± 0.10BC	27.68 ± 0.12D	42.44 ± 2.39AB	0.09 ± 0.00B	98.45 ± 8.19D	1.38 ± 0.01A

The data were presented as Mean ± Standard Errors. Different letters indicate significant difference among treatments (P < 0.05).

*DF1* weekly, *DF2* biweekly, *DF3* every 3 weeks, *DF4* every 4 weeks and *CK* ambient rain, *TP* Total phosphorous, *TN* Total nitrogen, *SOC* Soil organic carbon, *DON* Dissolved organic nitrogen, *MBN* Microbial biomass nitrogen, *AB* Aboveground biomass, *BD* Soil bulk density. The same below.

### Soil temperature and soil water contents

**Figure 1:**
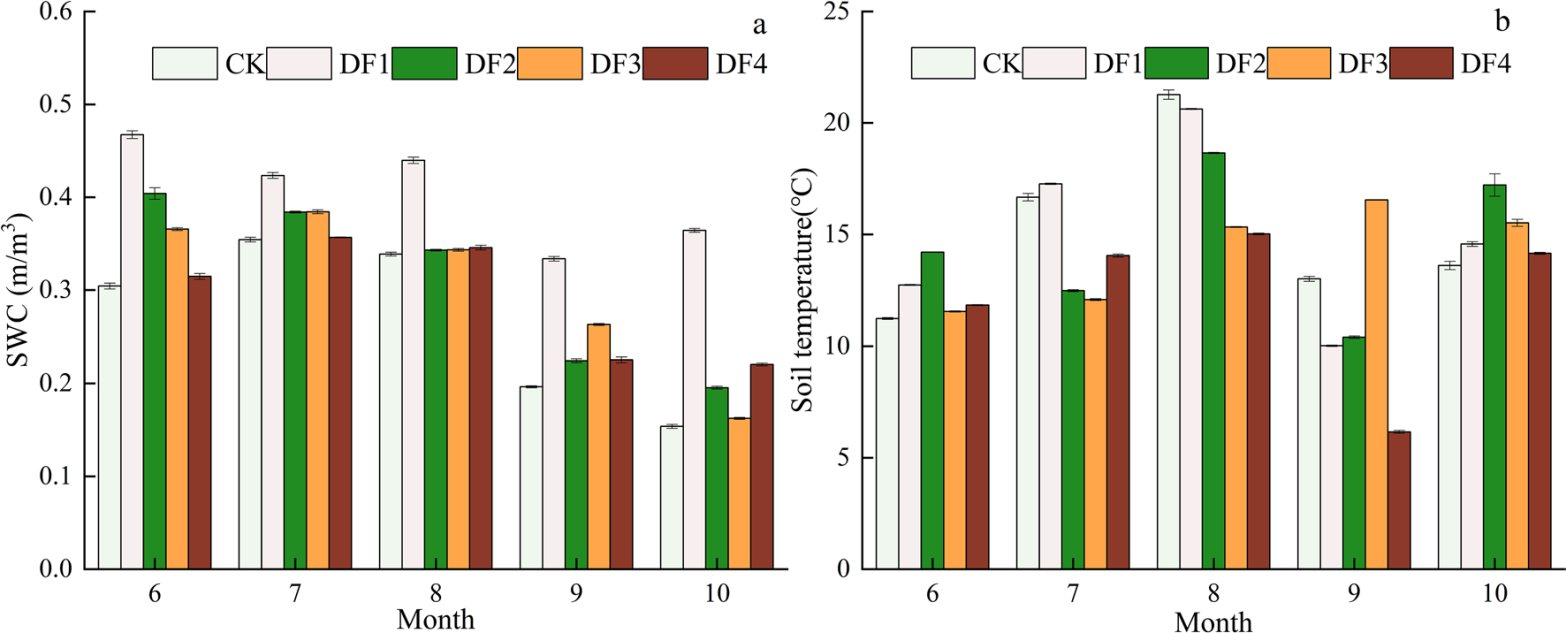
(**a**) Variation characteristics of soil water content; (**b**) Variation characteristics of soil temperature change; *DF1* weekly, *DF2* biweekly, *DF3* every 3 weeks, *DF4* every 4 weeks and *CK* ambient rain.

The changes in rainfall amount and frequency showed significant effects on the soil water contents and soil temperatures (Fig. 1). The soil water contents increased significantly with increasing precipitation frequency (P < 0.05). In addition, the soil water contents in DF1 and DF2 showed a decreasing trend with seasonal changes, while those in CK, DF3, and DF4 exhibited increasing–decreasing trends with seasonal changes. On the other hand, the soil temperature values of the five treatments showed consistent seasonal changes. However, significant differences in the soil temperature values were observed between the treatments (P < 0.05). The overall change in the soil temperature values exhibited an "N" shape over the entire vegetation growing season, showing the highest and lowest values in August and September, respectively.

### Soil mineral-N dynamics

**Figure 2:**
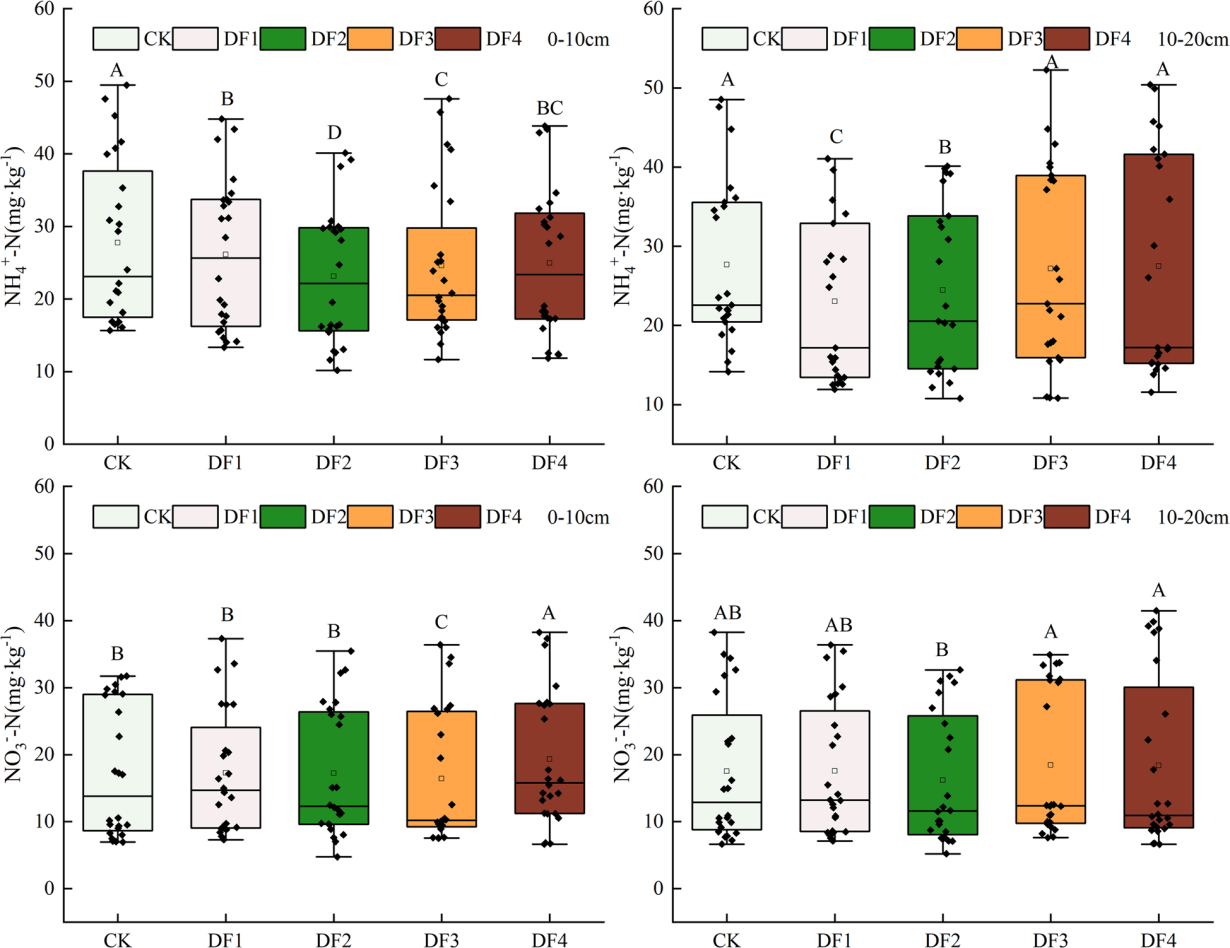
Rainfall amount and frequencies affects soil properties. Different letters indicate significant difference among treatments (P < 0.05). *DF1* weekly, *DF2* biweekly, *DF3* every 3 weeks, *DF4* every 4 weeks and *CK* ambient rain.

**Figure 3:**
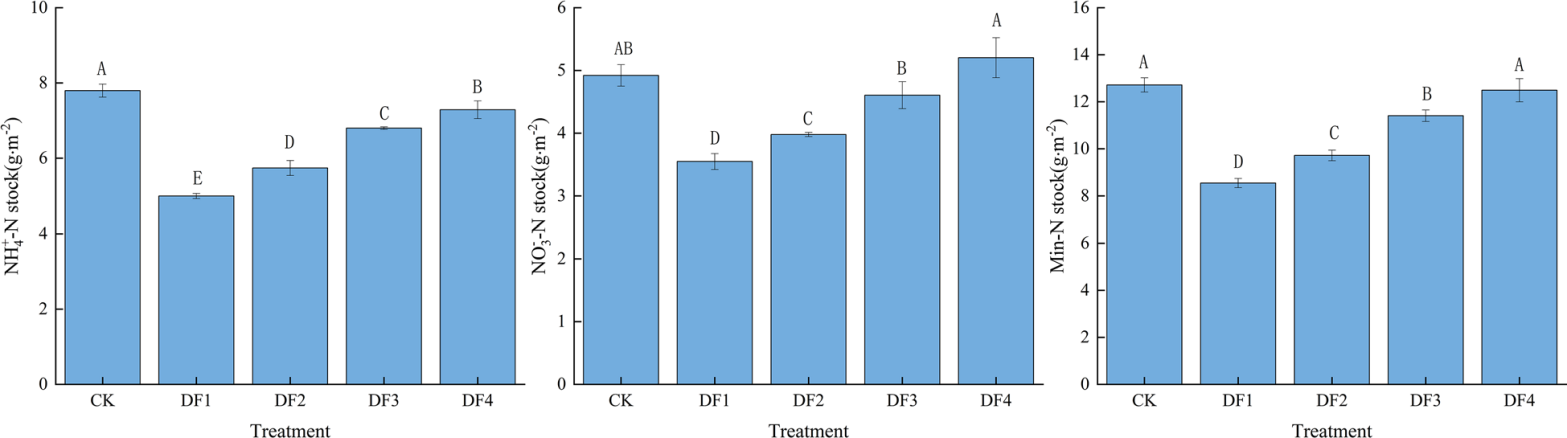
Rainfall amount and frequency on NH^+^_4_-N, NO^–^_3_-N and mineral N stock. Bars and error bars represent means and SD (n = 3). Different letters indicate significant differences in the mean value between different treatments.

The results indicated different effects of rainfall frequencies and amounts on the soil NH_4_^+^-N and NO_3_^–^-N contents in the Gahai wet meadows (Fig. 2). According to average values across the treatment scenarios, the NH_4_^+^-N and NO_3_^-^-N contents accounted for 59.4 and 40.6% of the total soil mineral N pool. These proportions were consistent with those observed in the soil layers. The NH_4_^+^-N and NO_3_^-^-N contents accounted for 59.1 and 40.9% of the total soil mineral N pool in the 0–10 cm soil layer, respectively. While in the 10–20 cm soil layer, the NH_4_^+^-N and NO_3_^–^-N contents accounted for 59.6 and 40.4% of the total soil mineral N pool, respectively. The increase in the rainfall amount and frequency decreased the soil NH_4_^+^-N contents. The NO_3_^–^-N contents were significantly higher in DF4 and lower in DF2 than those in CK. Furthermore, the interaction between the rainfall frequency treatments and soil depths exhibited a significant effect on the soil inorganic N contents (P < 0.01) (Table 2). The increase in the rainfall amount and frequency reduced significantly the mineral N stock (Fig. 3). In addition, it can be seen that the increase in the rainfall amount and frequency reduced the soil NH_4_^+^-N and NO_3_^–^-N contents, particularly in the 0–10 cm soil layer.

**Table 2:** Effects of rainfall amount and frequency and soil depth on soil inorganic nitrogen.

Sources of variation	df	NH_4_^+^–N		NO_3_^-^–N		Min–N	
		F	P	F	P	F	P
Treatments	4	39.017	< 0.01	12.153	< 0.01	19.797	< 0.01
Soil depth	1	12.363	< 0.05	0.320	0.578	4.639	0.045
Treatments × soil depth	4	27.917	< 0.01	7.664	< 0.01	9.330	< 0.01

### Soil N mineralization

**Figure 4:**
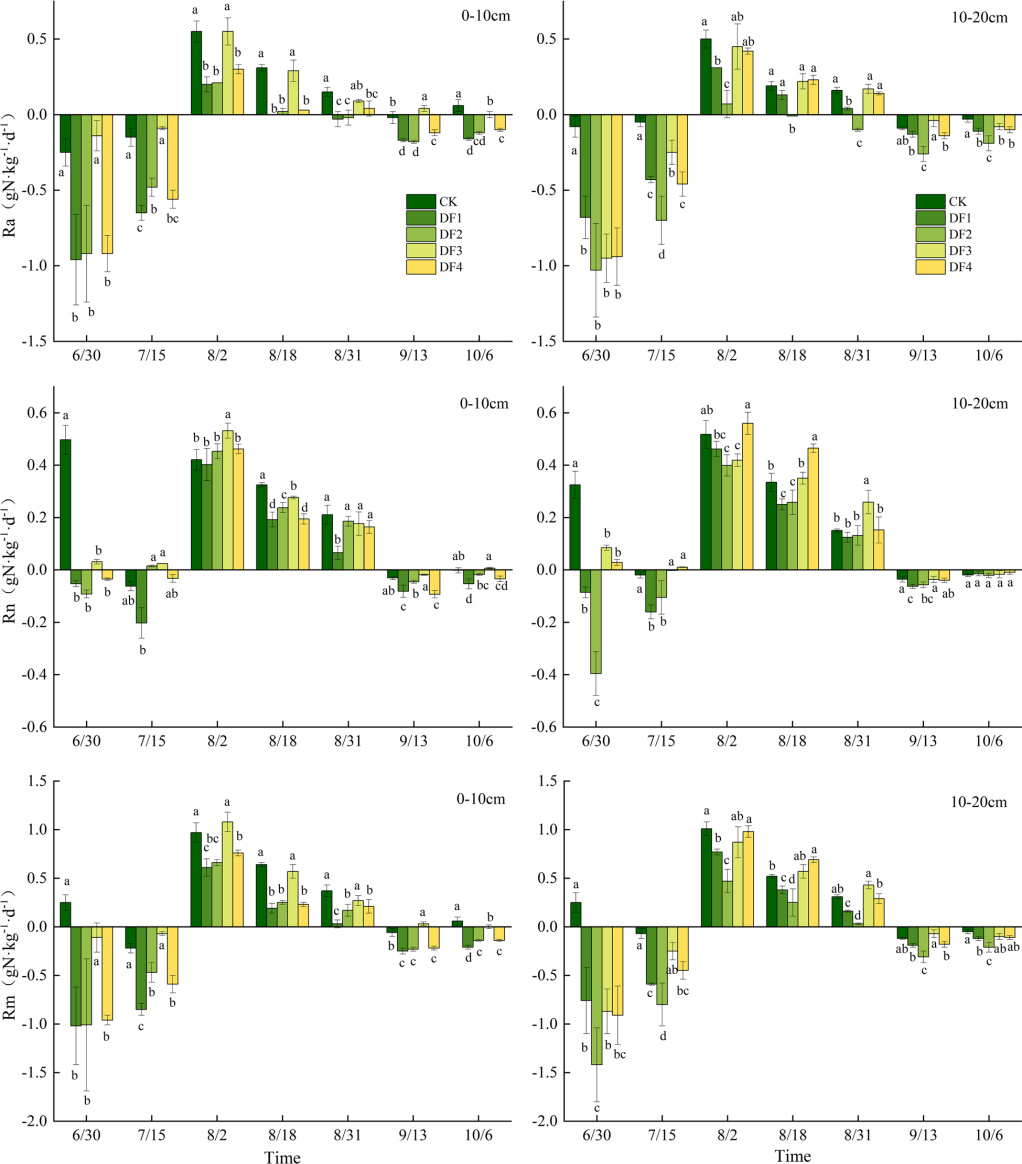
Seasonal dynamics of soil ammonification (Ra), nitrification (Rn) and mineralization (Rm) rates in plots with different rainfall amount and frequency. The values were averaged across 0–10 and 10–20 cm depths. Error bars are two standard errors of the means.

The average Ra, Rn, and Rm values obtained across the treatment scenarios were -0.099, 0.117, and 0.017 mg·kg^−1^·d^−1^, respectively. In addition, Ra and Rn were significantly and positively correlated with Rm (P < 0.01). However, the slope of the regression line between Rn and Rm (0.298) was lower than that between Ra and Rm (0.702) (Fig. 6). Therefore, Rm was dominated mainly by the ammonification process in the study site. Indeed, these patterns were consistent with the rainfall frequencies and soil depths and inconsistent with the different stages of the growing season (Table 2; Fig. 4).

The Ra, Rn, and Rm values of the five treatments exhibited obvious seasonal changes. However, although Ra, Rn, and Rm showed consistent seasonal trends, they were significantly different between the treatments (Fig. 4). For example, Rm showed an increasing–decreasing trend with the seasonal change, reaching the highest value in August 2019. Except for the CK treatment, the Rm values were negative in the June-July and September–October periods, indicating that soil N was in a net storage state during the non-growing season. In the DF3 experiment scenario, Ra and Rm in the 0–20 cm soil layer were significantly higher than those in the other experiment scenarios (P < 0.05). The interactions of the rainfall amounts and frequencies with the soil depths showed significant effects on Ra (P < 0.01), Rm (P < 0.01), and Rn (P < 0.05) (Table 3). Therefore, the obtained results demonstrated the importance of appropriate rainfall increases in improving Rm.

**Table 3:** Effects of rainfall amount and frequency and soil depth on soil mineralization rate.

Sources of variation	df	Ra		Rn		Rm	
		F	P	F	P	F	P
Treatments	4	43.044	< 0.01	16.593	< 0.01	39.130	< 0.01
Soil depth	1	0.737	0.402	0.183	0.674	0.181	0.675
Treatments × soil depth	4	7.522	< 0.01	4.422	< 0.05	6.713	< 0.01

### Relationships between soil mineralization rates and environmental factors

**Figure 5:**
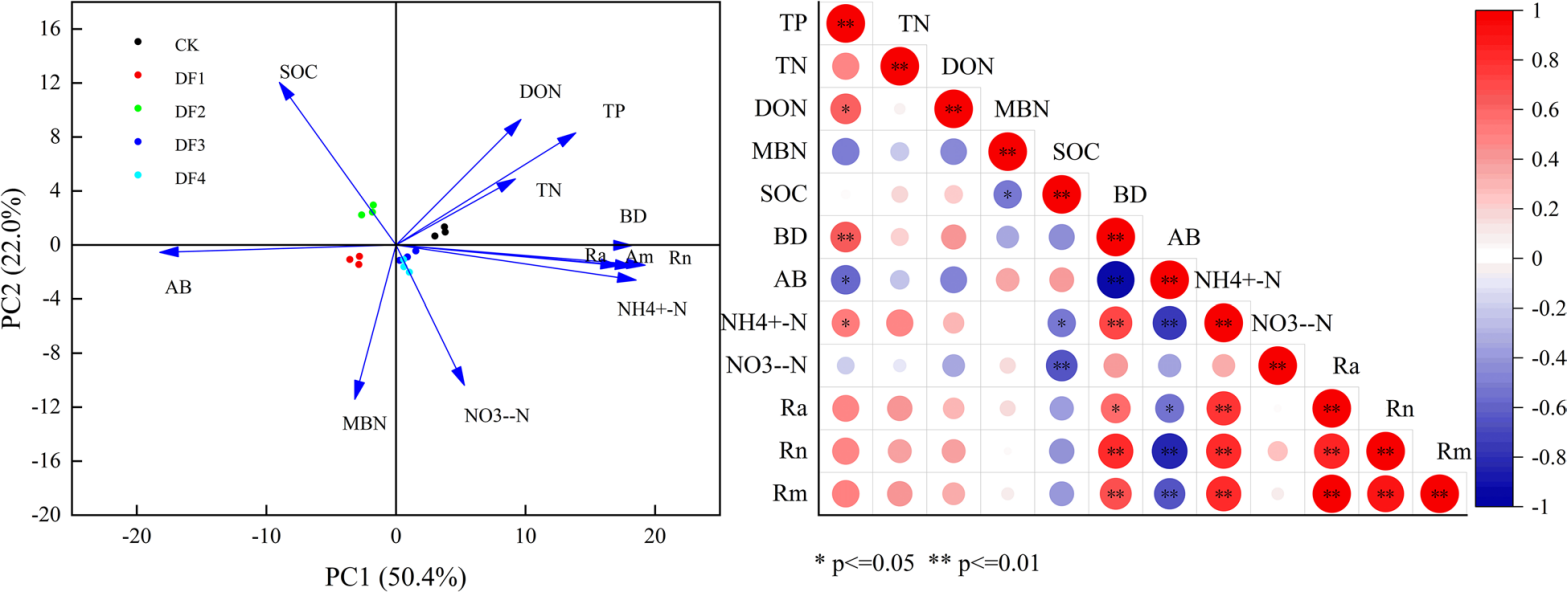
Principal component analysis of soil mineralization rate and physical and chemical properties of soil, and correlation between soil mineralization rate and soil physical and chemical properties. *Indicates significant at 0.05 level, **indicates significant at 0.01 level.

**Figure 6:**
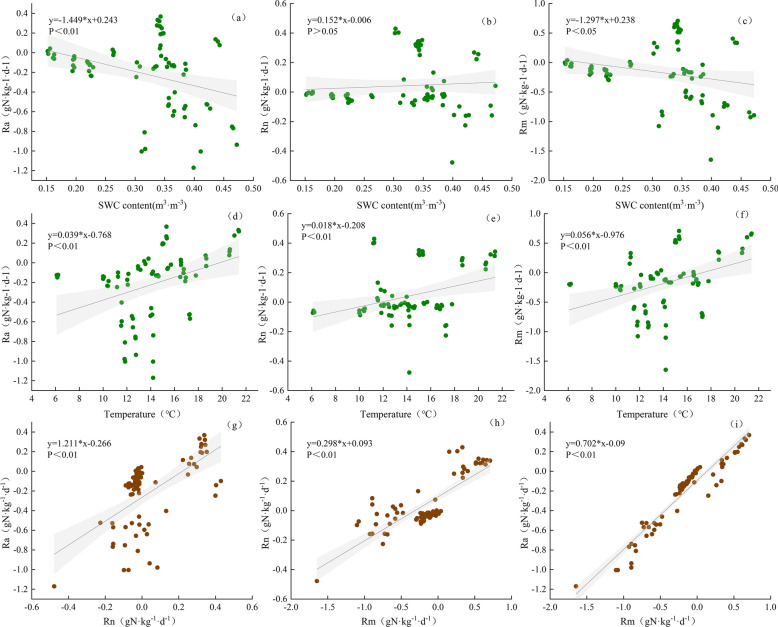
Relationship between soil mineralization rate and environmental factors.

In this study, the principal component analysis (PCA) was performed to reveal the relationships between the soil physicochemical characteristics and Ra, Rn, and Rm. According to the obtained results, the Rm values were significantly and positively correlated with BD, NH_4_^+^-N, NO_3_^–^-N, and soil temperature (P < 0.01) (Figs. 5 and 6). However, Ra, Rn, and Rm exhibited significant negative correlation coefficients with AB of -0.559, -0.826, and -0.662 (P < 0.01), respectively. Furthermore, Ra and Rm showed significant negative correlation coefficients with SWC of − 0.358 and − 0.235, respectively (P < 0.01) (Fig. 6).

## Discussion

### Soil mineral-N pool

Soil inorganic N forms (NH_4_^+^-N and NO_3_^–^-N) are derived mainly from the decomposition and transformation of soluble organic N by microorganisms. These N forms can be directly absorbed and utilized by plants. In this study, the NH_4_^+^-N and NO_3_^–^-N contents decreased significantly with increasing rainfall frequency (Fig. 3), which is consistent with the results of rainfall experiments conducted in semi-arid steppes of Patagonia^52^. This finding might be due to the positive roles of rainfall frequency and amount increases in increasing soil moisture contents (Fig. 1) and community species richness^53^, resulting in significantly high AB amounts (Table 1) and, consequently, enhancing the uptake of soil NH_4_^+^-N. Ammonium N (NH_4_^+^-N) is the main N form absorbed by plants due to its high solubility in the soil, increasing the chlorophyll-a content of plants and playing a key role in the leaf functional traits, thus increasing the AB amounts^54^. In addition, it was shown that rainfall variability significantly affects plant communities and their growth characteristics, as well as species compositions and diversity^55^. Indeed, precipitation frequency and amount increases can increase plant species richness within a plant community and result in ecological-niche divergence^55^. The positive correlation between species diversity and productivity can be explained by resource complementarity and sampling effects. Resource complementation effects suggest that species ecological niche differentiation and interspecific facilitation among plant communities with high diversity can improve resource utilization efficiency and, consequently, enhance community productivity^52^. Whereas the sampling effect indicates that the increase in the species diversity of a plant community results in a higher likelihood of including more productive species, thereby leading to higher community productivity and greater uptake of soil inorganic N and resulting in lower soil inorganic N contents^52^. On the other hand, the significant decreases in the soil NO_3_^–^-N contents with increasing rainfall frequency might be due to the occurrence of the leaching process, which can be promoted with increasing rainfall frequency. Indeed, NO_3_^–^-N cannot be adsorbed on the negatively charged soil colloids and mineral surfaces, resulting in a significant decrease in the soil NO_3_^–^-N contents^56,57^. In addition, the soil BD and soil water contents decreased and increased, respectively, with increasing rainfall frequency and amount (Table 1). The changes in the soil BD and soil water contents might inhibit soil microbial activity and, consequently, reduce the soil inorganic N contents^58^. In addition, the results of the present study highlighted the significant effects of the interaction between soil depths and rainfall frequencies on the soil NH_4_^+^-N and NO_3_^–^-N contents (Table 2). This finding might be due to the abundance of plant roots and microbial activity in the 0–20 cm soil layer in the wet meadow^59^, supplying more organic N into the soil through root decomposition.

### Effects of soil moisture regulation on soil N dynamics

Rainfall can significantly affect the N cycle in terrestrial ecosystems and physically affect the microbial-mediated N mineralization process^11^. On the one hand, rainfall changes can directly affect soil water infiltration and N volatilization^60^. On the other hand, rainfall can affect N transformations, root uptakes, and microbial utilization, thereby directly or indirectly affecting the soil N cycle process^61^. In this study, Ra, Rn, and Rm were negative in all the treatment scenarios from June to July and from September to October, indicating N immobilization by soil microorganisms. In addition, NH_4_^+^-N might be stored by N-limited microorganisms or converted into NO_3_^–^-N by nitrifying bacteria. This also implies that microbial growth in the wet meadow soils may not be carbon-limited. The soluble organic N stock in the soil is not conducive to microbial growth and respiration^62,63^. In contrast, Ra, Rn, and Rm were positive in August, which might be due to the gradual increase in the soil temperature, reaching the highest value in August (Fig. 1). In this period, soil microorganisms can exhibit high activity and strong competition for carbon. As a result, the availability of carbon may become a limiting factor for soil microbial growth. Indeed, soil microorganisms can utilize soluble organic N to meet their growth requirements, resulting in a net release of N. This suggestion is consistent with the high and significant positive correlation between the soil temperature and Rm values observed in this study (Fig. 6). In early July, the Ra, Rn, and Rm values of CK were significantly higher than those observed in the other treatment scenarios. This result was probably due to the first applied irrigation rate in late May when high soil water contents and low soil temperatures were observed following rainfall events, thereby resulting in a faster increase in soil temperature and higher Rm in CK compared to the other treatments. This study also found significantly higher Ra values in the DF3 treatment scenario than those in the remaining treatments, which might be due to the increased soil soluble nutrient contents and accelerated soil microbial activity with increasing rainfall and frequency^63,64^. Indeed, the increase in soil microbial activity can promote the decomposition of organic matter, thus increasing the Ra values. However, high soil water contents exceeding the threshold can enhance the anaerobic conditions in the soil, thereby preventing soil microbial oxidation, which is detrimental to litter decomposition and, consequently, reducing Ra^65^. Ammonium (NH_4_^+^-N) availability and oxygen concentrations are the main factors affecting the nitrification process^66^. The results of the present study revealed a decrease in Rn with increasing rainfall frequency, which may be due to two reasons: (1) the decrease in the soil NH_4_^+^-N contents with increasing rainfall was not conducive to NH_4_^+^-N migration to nitrifying bacteria, thus limiting the availability of soil NH_4_^+^-N; (2) High soil water contents can lead to low dissolved oxygen concentrations in the soil solution^67,68^, thereby inhibiting the nitrifying bacterial activity and leading to a decrease in Rn. In this study, low Rm values were observed during the growing season, suggesting limited soil organic mineralization and potentially representing a fraction of the effective natural flux of organic matter. Indeed, since the plant root system and mineralization action of mycorrhizal fungi need to be considered when using the buried PVC pipe method, Rm can be underestimated in the presence of plant root systems in soils. In addition, the increase in the soil water content within a certain range can promote N mineralization. However, the appropriate soil water content range for soil N mineralization varies among ecosystems, depending on several factors, including soil type, soil texture, and microbial community^69,70^. In this study, the Rm values of the DF3 treatment scenario were significantly higher than those observed in the remaining treatment scenarios. This result might be due to the decreased soil permeability and microbial metabolic activity with increasing rainfall amount and frequency, resulting in reduced Rm values^71^. Moreover, the low Rm values might be due to slightly acidic rainwater, which can decrease the soil pH values and, consequently, increase the fungi-to-bacteria ratio, as fungi are characterized by a higher tolerance to high H^+^ concentrations than bacteria due to their thick and interconnected peptidoglycan cell walls^72^. Soil Rm can only be enhanced under relatively suitable soil moisture conditions.

## Conclusions

In this study, we investigated the effects of rainfall amount and frequency on soil mineral N and net N mineralization during the growing season in the Gahai wet meadow. Our findings demonstrated the significant effect of rainfall on driving the soil N turnover process in the alpine wet meadow. According to the obtained results, the increased rainfall reduced soil mineral N stocks. However, NH_4_^+^-N was the dominant N mineral form in the soil. The highest Rm value was observed in the DF3 treatment scenario during the vegetation growing season. In addition, the Rm values were significantly and positively correlated with soil temperatures and negatively correlated with soil water contents and AB amounts. The results of the present study provide further insights into the N cycle responses to changes in rainfall amount and frequency, as well as into the underlying mechanisms of the rainfall amount and frequency effects on plant and ecosystem functions. However, soil N turnover processes, such as plant root N uptakes and soil gaseous N emissions, were not considered in this paper. We suggest, therefore, further related studies in the future, taking into account plant root N uptake and gaseous N emissions.

### Supplementary Information

Supplementary Information.

## Data Availability

All data generated or analysed during this study are included in this published article (and its Supplementary Information files).
